# A Clinical and Practical Treatise on the Diseases of Children

**Published:** 1845-07

**Authors:** 


I. TRAITÉ CLINIQUE ET PRATIQUE DES MALADIES DES ENFANS. Par MM. *Rilliet* et *Barthez*. Tom. 3. 8vo. pp. 2370. Paris 1843.

A Clinical and Practical Treatise on the Diseases of Children. By MM. *Rilliet* and *Barthez*.

II. MANUEL PRATIQUE DES MALADIES DES NOUVEAUX-NÉS ET DES ENFANS A LA MAMELLE. Par *E. Bouchut*. Small 8vo. pp. 610. Paris, 1845.

A Practical Manual of the Diseases of New-Born Infants and Children at the Breast. By *E. Bouchut*.

III. PRACTICAL OBSERVATIONS ON THE DISEASES MOST FATAL TO CHILDREN. By *P. Hood*. 8vo. pp. 231. Churchill, 1845.

WE have long intended to present our readers with a succinct analysis of the contents of the important work placed first upon the above list, but have been prevented undertaking the somewhat laborious task by the pressure of other matters. The contributions of the French press to this branch of medical literature have been of late years numerous and valuable, but have chiefly related, as in the works of Billard and Valleix, to the diseases of new-born infants. It is the object of MM. Rilliet and Barthez to present a detailed account of the maladies affecting children aged fifteen months and upwards, and the complete manner in which they have carried it out must render their work a high authority for a long period to come. Paris offers in her large Foundling and Children's Hospitals opportunities for the pursuit of these investigations, which do not exist in this country. The absence of such establishments is not, however, a subject of unmingled regret; for there can be no doubt, as we observed in our notice of M. Billard's treatise, (Med.-Chir. Rev. Vol. 32), that much of the disease found within their walls, is generated by the assembling together of such large numbers of these little creatures; and the modifications produced in the nature and signs of the various affections by the operation of this circumstance, must prevent our too rigorously applying the conclusions derived from such a source to the exigencies of private practice. The authors have for several years laboriously cultivated this ample field of research with a view to the publication of the results. Besides the minute pathological statements and able diagnostic descriptions, so characteristic of the French school, we find a better acquaintance with the writings of others, and a more accurate statement of therapeutical indications than are usually displayed by our continental brethren.

M. Bouchut's Manual relates to younger children, and is evidently the work of a judicious observer, qualifying as it does, in some instances, the hasty conclusions arrived at by preceding writers. It is the result of a two-years' study in the wards of Professor Trousseau.

Mr. Hood's work is about as unfit a one to put into the hands of a young practitioner as those above-mentioned are suitable for this purpose. The confidence of the tone assumed and the error of the doctrine taught, alike render it dangerous. Fortunately, adducing neither fact or argument worthy of the name, its authority is not likely to become very great. A complete *hæmatophobia* has seized the author, and he sees in the child but a bloodless, sensitive, irritable, little creature, whose maladies must ever be aggravated by depletion, however guardedly employed. He does not admit of the possibility of the use of the lancet, or the application of a single leech being admissible; and most of the few cases he alludes to are examples of his rescuing the victims from the consequence of more active procedures. The diseases of children ordinarily considered as inflammatory, and treated by depletion, such as bronchitis, pneumonia, croup, convulsions, hydrocephalus, &c. depend upon, according to Mr. Hood, the presence of a state of "irritation," in which blood-letting is worse than useless. The readers of this Journal need not be told how often we have protested against indiscriminate and excessive depletion at any age, much more that of childhood; and the attention of the medical observer has been forcibly called of late years, by several talented men, to the existence of conditions of the infantile economy which forbid debilitating measures of any kind. But the proscription of bleeding, local or general, under all circumstances, and the rash recommendation of opiates, tonics, and stimuli, in conditions of the system ill-fitted to endure them, were reserved for Mr. Hood; who does not find it requisite to embarrass himself with diagnostic minutiæ, and consequent variety in therapeutical indications; but having determined that children's diseases arise from and consist only in "irritation," simplifies his practice accordingly. This book should at least bring consolation to those practitioners who, observing the latter stages and post-mortem appearances of some of these affections, have felt poignant regret that more active measures were not instituted at an early period!

Our attention will be chiefly directed to the work of MM. Rilliet and Barthez, adding any illustrative particulars that may offer themselves in that of M. Bouchut. Both works contain some introductory observations, a few of which it may be as well to glance at.

1. *The Physiological Condition of Children.*—In children the *lymphatic* temperament especially prevails, which disenables them from offering strong resistance to injurious agencies, such as errors of diet, cold, impure air, &c.; but as they advance in age their susceptibility diminishes, their flesh becomes more firm, and the diffusion of heat is more uniform. The *pulse* being in children so rapid, their susceptibility to cold must depend upon some imperfection of hæmatosis. M. Trousseau found the numbers vary in children aged less than 21 months from 96 to 160, the mean number being 137 in the 1st and 2nd month, 128 from the 2nd to the 6th month,

120 from 6 months to a year, and 118 from 12 to 21 months. After the third month the pulse of girls commences to be more frequent than that of boys. Sleep has a remarkable effect, the number then diminishing 15 or 20 beats. Fear and other causes produce great acceleration, and the mere rapidity of pulse is therefore seldom an indication of treatment in the child, unless accompanied with fever. The *inspirations* vary from 20 to 32 in children from 2 to 5; from 20 to 28 in those from 6 to 10 years of age. They are regular, full, and noiseless, and during sleep in young children are often so deep as to amount to sighing. In these young subjects, too, the respiratory movements are often irregular or intermitting without any derangement of health. The normal form of the *thorax* and *abdomen* must be borne in mind. A depression which extends from the xyphoid cartilage around the lateral portions of the chest (the false ribs being at the same time expanded), owing to these yielding more readily to the attachments of the diaphragm, and the small capacity of the pelvis, cause a remarkable projection forward of the abdomen, which in very young children may be mistaken for disease. After some years the pelvis becomes enlarged, the margins of the ribs more resisting, and the liver diminished in size—so that the superior and inferior abdominal organs making no projection, the marked boundary between the two cavities ceases to appear.

*Auscultation.*—The chest is more sonorous in the child than in the adult, and the vesicular murmur, which is also more intense, is only heard during inspiration. MM. R. and B. prefer mediate percussion by means of the finger and immediate auscultation, as the stethoscope frightens some children and pains others. In ricketty children, and others, in whom the chest is deformed, the examination is made more easily by having the child held to the ear horizontally in the arms of the nurse. The maximum of sound is perceived from just below the clavicle to near the nipple anteriorly, and in the intra-scapular region behind. M. Bouchut observes that, however true it is that the respiration is louder (*puerile*) in children more than two years of age, such is not the case in *children at the breast*. On the contrary, probably from the air not completely dilating the vesicles, there is very little sound indeed. So too in a healthy infant percussion is dull, being however sonorous if the child is very thin. The sound elicited even from the same child is liable to great change and variety, and is not to be relied upon.

2. *On the Examination of Sick Children.*—MM. Rilliet and Barthez give detailed explanations of the plans they followed in collecting the particulars of the various cases which came before them. They justly observe, without some mechanical aid of this kind, it is impossible for the medical officers of large institutions to record these with the requisite accuracy and minuteness. For each patient four sheets of paper are provided, one of which is dedicated to his history and state on admission, the second is a record of his progress and symptoms from day to day, and the third is a register of the results of the autopsy. The fourth forms a cover for the others, having written upon it a *resumé* of the particulars they detail. In the margin of each of these sheets every particular concerning which inquiry has to be made is *printed*, thus saving valuable time to the narrator, and preventing his overlooking any important point. One can judge upon a small scale how convenient this plan must be, by comparing the much less trouble it takes to reply to the medical queries of an insurance office, than it would do if the statement had to be made unaided by these. Examples of the tables are given, to which we refer those of our readers who are connected with large institutions.

3. *On the Administration of Medicinal Substances to Children.*—The authors observe that, although in the course of their work they frequently recommend active medicines, yet that they agree with Henke, that if ever expectant medicine is justifiable, it is so in some of the diseases of children. Many of the functional disturbances do not require active drugs, and in others the diagnosis is but imperfect. Practitioners should remember too that, owing to the carelessness of attendants and the obstinacy of children, all they send is never administered. But we may ask, does this not often arise from the even yet continued practice of sending far more medicine than is required—a dangerous practice, for the parent quite unable to give all, is as unable to determine which and what portion is essential. The obstinacy on the part of children is chiefly manifested in their slighter affections.

MM. Rilliet and Barthez also deservedly condemn the *routine* practice which always treats the same disease in the same manner, without due consideration of the actual condition of the patient, the stage of his disease, or the hygienic circumstances which surround him. In almost all diseases, a period arrives in which the consideration of the general condition of the patient should predominate over that of the organ specifically affected.

*Modes of Administering Medicine.*—Giving children medicine by the *mouth* is not always practicable, and when other means suffice, they should not be irritated by the attempt. Where it is essential, the nose should be pinched with one hand, and the spoon introduced completely into the mouth with the other. Is sufficient pains usually taken to disguise the nauseous flavour of medicines? *Injections* are much used in France, for the conveyance of other substances besides laxatives. The dose required is often *less* than when given by the mouth. The substance is more readily absorbed if the bowel is first emptied and the mucous membrane cleansed by means of an emollient glyster thrown up just before. So too the action of an injected substance is often extended by following it with an abundant watery enema. The authors much approve of administering medicines by rubbing them into the *skin*, believing this very preferable to the endermic method. The skin should be well cleaned with warm water before the frictions are employed, which require repetition several times in the twenty-four hours. The inner part of the thigh is a very eligible site for absorption, or a piece of wool smeared with ointment may be retained in the armpit by means of a handkerchief. M. Prevost says, the facility with which any ointment is absorbed is much increased by adding 1 part of *ung. hydr.* to every 32 parts. *Baths*, simple or medicated, are highly useful, the authors usually employing them at from 26 to 28 Reaumur. A child may frequently be retained in a simple warm bath for one or two hours, but in a medicated one not longer than from 15 to 45 minutes. *Foot-baths* are seldom used, and Hufeland says that steeping the feet with cloths dipped in hot milk forms a good substitute—inducing copious transpiration. *Hand-baths* are frequently used, and are very convenient, as the child need not be removed from its bed. *Cataplasms* and *fomentations*, so frequently used for children, require still more frequent renewal than they do in adults, to prevent chilling. *Blisters* are seldom used by MM. R. and B. in consequenee of the irritation they so often give rise to; and are especially to be avoided in very thin children, during the course of convalescence and chronic or enfeebling diseases, and during epidemics. Notwithstanding the observations of M. Bouchut relate to much younger children, he is a warm advocate of the utility of blisters applied for a brief period. Although the authors are more bold than their predecessors in this respect, they still have a very unfounded dread of *purgatives* in many of the diseases of children.

In their preliminary remarks MM. Rilliet and Barthez remark, that the *diseases of children* seldom pass through their various stages without becoming *complicated* with, or followed by, other morbid affections. On this account they might be classed in three categories: diseases which almost always appear during the existence of good health; those which are nearly constantly the consequence of a prior morbid state; and those which sometimes occur spontaneously and at others succeed another affection. In fact, the due consideration of the *primary* or *secondary* nature of the child's disease is of vital importance. The more diseases resemble each other the greater is their tendency to complication. Thus, if the primary affection be a phlegmasia, secondary or tertiary phlegmasiæ easily follow. The presence of dropsy or tubercle in one organ is usually accompanied by its existence in others. When the secondary disease is different in nature from the primary one, the latter has acted as a stimulus to its production. Thus tubercle may give rise to an inflammation of the organ in which it is deposited, and pertussis may develop a bronchitis or pneumonia. Although the severity of diseases is usually augmented by complication, they occasionally, when of a different nature, produce a contrary effect. Thus, scarlatina or small-pox occasionally cure tubercle: and some of the phlegmasiæ relieve some of the neuroses. If the affections are of a similar nature, the primary one will only be relieved when no diathesis prevails, in which case pneumonia, *e. g.* may cause the disappearance of diseases of the hairy scalp.

The distinction of diseases into *acute* and *chronic*, is not always so easily made or so fundamentally important as in the adult. The diseases of children often assume an intermediate form—frequently in consequence of a continued succession of morbid conditions. The disease may be acute as regards its duration and the nature of the lesions it gives rise to, but chronic in the sympathetic disturbance which it excites. When an acute disease attacks a feeble infant and vigorous re-action is impossible, it may be termed *cachectic*.

The various affections are treated of under eight separate divisions, viz. Inflammations, Dropsies, Hæmorrhages, Gangrenes, Neuroses, Continued Fevers, Tuberculizations, and Entozoaires. This is a very faulty classification, giving rise to the separation of diseases possessing strong affinities of nature and locality, and leading to needless repetitions and omissions. Still, as our object is simply to place before our readers some of the principal facts, we do not think it necessary to depart from the order in which they here are detailed.

### Class I.—Phlegmasiæ.

Among the preliminary observations, there are some very good ones upon the various types under which inflammation presents itself in the child. Thus we may have the well-marked acute form in which the local symptoms and general re-action are well manifested, and a cure quickly results under antiphlogistic treatment, especially if the inflammation be not secondary. Other cases exist, acute in their progress and fatal in their lesions, but which are not accompanied by a degree of re-action proportioned to their gravity, and against which antiphlogistics employed alone are much less powerful. M. Bouchut remarks upon the absence or irregularity of the febrile action in young infants suffering from phlegmasiæ. An extract we shall give concerning what MM. R. and B. term the cachectic type of inflammation is worthy of notice.

"In this case the child is feeble and sinks down in its bed; its eyes are hollow, its skin dry, earthy, and yellow, its emaciation excessive; and its face covered with wrinkles like that of an old man. The almost lifeless muscles are seen to make scarcely any projection beneath the skin, the emaciation of the middle portions of the limbs giving their articular extremities an appearance of morbid tumefaction. Or the face may be pale, waxy, and œdematous, and the skin flabby, thin, and almost diaphanous. The limbs are infiltrated and the fleshy portions soft—the child in fact presenting the appearance of advanced cachexia. On touching the skin we find it cold, and can scarcely feel the small thread-like pulse. Food, even not of a tempting kind, is often taken with avidity, and a colliquative diarrhœa is commonly present.

"The preservation of the appetite and the general debility which is present, may prevent at first the belief that inflammation is present: but an attentive examination reveals the existence of an extensive, dangerous, acute inflammation, although it does not manifest itself by any of its proper external symptoms. Examination after death exhibits the traces of the inflammation which had been detected during life, as well as of other phlegmasiæ which had escaped attentive exploration, and oftentimes the chronic lesions which had given rise to the cachexia.

"Such is the appearance which some of the phlegmasiæ present, especially in early childhood. To treat such by antiphlogistics and debilitants would only be to hasten or to cause a fatal termination; while, by the use of tonics, or even of appropriate hygienic means, we may prolong the days of the child, or even effect a cure. We term this state *cachectic* rather than chronic; because the phlegmasiæ which are in the adult anatomically speaking chronic, are, in the child, sometimes chronic, sometimes acute.

"This different expression of diseases whose anatomical characters are identical, is so important, that its reality cannot be too much insisted upon. Two different causes (excess of tonicity and atony) produce the same effects; and if, guided only by the anatomical lesion, you attack the disease by the same remedies, you exasperate it in the one case and relieve it in the other. The treatment of phlegmasiæ in children should derive its indications rather from the symptoms than the pathological anatomy."

Inflammations are seldom *solitary* in children, and the serious consequences of a combination are thus stated:—

"A child attacked with bronchitis soon gets a pneumonia—to this pneumonia succeeds an enteritis or *vice versa*. In fact, inflammations succeed each other with the greatest facility, or rather they proceed simultaneously, aggravating rather than relieving each other. This remark, which admits of few exceptions, is of such therapeutical importance, that we may dwell upon it awhile.

"Suppose that an inflammation, developed at first in an organ of little importance, afterwards appears in one that is more so; the condition of the patient becomes absolutely worse, while sometimes the original inflammation disappears, at others it remains stationary or becomes increased. Thus, a child having impetigo of the scalp, is seized with pneumonia. The impetigo disappears or remains stationary, and the child is in a more serious condition. A child suffering from diphtheritis, or only bronchitis, is seized with pneumonia: the first disease remains stationary or augments, and the child's condition is worse. So that an inflammation of an organ may disappear, remain stationary, or increase under the influence of another inflammation developed in a more important organ. But it is to be well observed that the disappearance of the first inflammation is the exception, and that the general condition always becomes worse.

"The reverse question is an important one. An inflammation exists in an organ essential to life, what happens when another inflammation develops itself in an organ of less or about equal importance? We reply, that the first inflammation will never, or hardly ever become diminished, and may be sometimes increased; and that the second inflammation will prove but an additional cause of death. Thus a child has pneumonia, and is seized with enteritis. The two diseases may proceed simultaneously, perhaps aggravating each other; but the enteritis will not cure the pneumonia. It is the same if an erysipelas or cutaneous eruption appears, *or if cutaneous revulsives are applied*. A sinapism or blister is often a painful excitation powerless for the cure of the internal malady, which pursues its course in spite of, or even aggravated by, this new phlegmasia.

"Phlegmasiæ may also become complicated with any other affection, whose gravity adds to theirs. Thus we see specific inflammations, and any of the diseases mentioned in our general table, may combine with the phlegmasiæ. Measles, small-pox, dropsy, hæmorrhage, tubercle, diseases of every kind in fact, may develop themselves either in the course of, or at the end of a phlegmasia—producing that entanglement and rapid succession of diseases, the source of the immense mortality of children."

### *Bronchitis.*

*Pathological Anatomy.*—In 174 autopsies, in which lesions characteristic of bronchitis were observed, *redness* of the mucous membrane existed in 143, and in 34 was found united with other lesions. The bronchitis was usually double (in 90), and when single (53) existed upon the right or left side in equal proportions. It was frequently confined to the inferior lobe, especially on the left side, but in the great majority of cases occupied the entire lung. In the 174 autopsies *dilatation of the bronchi* was found 74 times, such dilatation existing oftenest on the right side, and more frequently confined to one lobe than not. In 16 cases *cellular dilatation* (*en vacuoles*) was found; and in 17 out of the 174 cases *vesicular bronchitis* existed, being more frequently single than double, and occupying especially the left inferior or the right middle lobe. In 10 of the cases *false membranes* were found in the bronchi.

Each of these changes is described at considerable length. The authors believe that sufficient attention has not been paid to *dilatation of the bronchi* as a consequence of bronchitis in children. Sometimes the tubes are found dilated in their course, and at others only at their extremities. The dilatation of these latter into little ampullæ, which is never found where the bronchitis has not continued for 21 days, sometimes gives rise to the appearance of small abscesses resulting from pneumonia. These M. Bouchut states are rarely seen in children at the breast, but in their place small ecchymosed spots are found as soon as the bronchitis becomes general, and is about to pass into the state of pneumonia. In chronic dilatation the walls of the dilated tubes become thickened, and the pulmonary parenchyma in part disappears, giving the section of the lung the appearance of that of a cheese having a great number of "eyes." *Vesicular* or *capillary* bronchitis is seldom found without accompanying inflammation of the parenchyma. Acute bronchitis is rarely found in a child less than five years old without accompanying pneumonia, the latter being the secondary disease, although the two may be developed simultaneously.

The authors state primary bronchitis to be a much rarer disease than it will be found to be in private practice. Thus of 115 cases of uncomplicated bronchitis, 21 only are set down as primary, the remaining 94 having become developed during the course of other diseases. M. Bouchut believes the primary form of the disease to be as frequent or more so than the secondary, but being less serious and fatal, comes less under the cognizance of the hospital physician. Age exerts considerable influence in predisposing to bronchitis—the younger the child, the less being the liability to it. Thus of the 115 cases, 37 were less, and 78 more than five years of age. Primary bronchitis occurs most frequently in girls, and secondary in boys, which is explained by the greater frequency of typhoid fever among the latter, a disease furnishing the greatest number of secondary bronchites. Measles, pertussis, and rickets, are other diseases during which its supervention is common.

The *treatment* in only moderately severe cases consists in the use of abundant ptisans, James' powder, emetics, and in older children a few leeches. When the cough is distressing it is much relieved by belladonna, and when it continues the only remaining symptom, by flowers of sulphur in small doses. In severe bronchitis a more active depletion may be required, but the authors caution against the rash and too often repeated employment of this, exhausting the powers of the child, and disenabling it from bearing up against the impending asphyxia frequently dependent upon large accumulations of secretions. After moderate but effectual depletion *emetics* must be much relied upon; and, if the child is not seen until a late period of the disease, they must frequently be substituted for depletion of any kind. They must be continued as long as the chest is oppressed by abundant secretions: but they are much less efficacious at a late period of the disease, seldom then exciting the stomach, abdominal muscles, &c. to vigorous action, being often either not returned at all, or as if by simple regurgitation. At this latter period successive sinapisms or blisters not applied long enough to cause vesication, are very useful remedies, by reason of their *revulsive* effect. Cruse has found in this adynamic stage the use of musk or serpentary, and the employment of warm aromatic baths, highly useful. The child should not be allowed to be much on its back, but retained chiefly in the sitting posture or on its side, in order to facilitate the expulsion of accumulated secretions. In *chronic* bronchitis the diet must be good, and tonics with an occasional emetic prescribed; while the cough is best treated with some resinous balsam, as tolu, &c.

M. Bouchut, speaking of the treatment of this disease in children at the breast, recommends the employment of warm cataplasms or hot flannel compresses to the chest, emetics, and afterwards, if pneumonia seems supervening, the temporary application of a blister as large as the chest itself, or the use of the croton oil ointment. The loss of blood he considers as very rarely advisable.

*Pneumonia.*

*Pathological Anatomy.*—The *Lobar Pneumonia,* as seen in the adult, is not the form usually met with in children. This is the *Lobular* in which certain lobules are inflamed, those surrounding them continuing unaffected. When the inflammation is perfectly circumscribed the authors term it *mammelonnated,* in which small spherical kernels of hepatized lung, varying in number and size, are scattered amidst the healthy texture. In these, the formation of abscess is by no means rare, still continuing in their isolated condition, or, in other cases, involving by their junction a part or the whole of a lobe. They are more commonly found in one lung than in both, and in children less than six years of age. *Partial lobular pneumonia* is distinguished from the foregoing by the line of demarcation separating it from healthy structure being less precise. Its volume is usually more considerable, and its form more irregular. While the centres of these inflamed portions are passing into the second stage, the circumference may be in the first, and joining with other inflamed portions until a state of *general lobular pneumonia* is induced. All these, as well as lobar pneumonia and vesicular bronchitis, are in fact but varieties in extent of the same phlegmasia, of which vesicular bronchitis may be represented as the first, and lobar pneumonia as the last, link of the chain. These various stages are not however necessarily passed through; for there can be no doubt that pneumonia may occur in children without prior affection of the bronchi. The entire of lesions reported amounted to 314, viz. 70 presenting the mammelonnated, 140 the partial, and 104 general pneumonia; but as in 111 of these cases there was a combination of the different forms, the autopsies were really but 203, of which number only five cases were noted in which the pneumonia was not double. The change described by M. Rufz under the name of *carnification,* in which certain lobules or even a whole lobe are converted into a dense fleshy-like body, much resembling the lungs of a fœtus which has never breathed, was met with 42 times, either in one (generally the right) or both lungs. The authors regard it as a species of chronic pneumonia, and indeed as the only form of this ever found in non-tuberculous children.

*Of the Complication of Pneumonia with Bronchitis,*—MM. Barthez and Rilliet thus speak:

"In the great majority of fatal cases of the pneumonia of children bronchial inflammation is found. We have verified it in all its forms and degrees, from the state of simple injection with mucous secretion to acute dilatation of the bronchial tubes filled with purulent fluid or pseudo-membranes. We need not repeat the anatomical characters of bronchitis, and will only observe—(1). That the form which coincides with pneumonia almost always is seated in the small tubes. (2). In the great majority of cases, it co-exists with one of the forms of lobular, and rarely with lobar, pneumonia. (3). Bronchitis with dilatation is found almost exclusively in infants who have died from the lobular, and hardly ever in those who have died from the lobar form. (4). The bronchitis exists almost always at the circumference or centre of the hepatized portions, but may also be found elsewhere. (5). Dilatation of the bronchi is very frequent in the carnified tissue."

*Respiratory Movements.*—Even at the commencement of the disease these are much accelerated, varying from 40 to 60, according as the child is younger. As the disease augments, they rise to 70 or 80 in the younger, and 50 or 70 in the older; and even when a cure is eventual, this rapidity never disappears before the sixth, and sometimes not until the 12th day. Changes in its character and rhythm are observed only in about half the cases, and do not seem always dependent upon the severity of the disease: but MM. R. and B. have remarked that inequality in the movements, and especially an interrupted character, is almost always found connected with pneumonia of the apex in young children. In *secondary* pneumonia the acceleration of the respiration is often of very long duration and dependent upon the nature of the primary disease. In some cases of broncho-pneumonia the dyspnœa becomes as excessive as in suffocative catarrh. In *cachectic* pneumonia there is far less acceleration. M. Bouchut has some interesting remarks upon this subject.

"The acceleration of the respiration always manifests itself as soon as the commencing pneumonia is established, reaching from 60 to 80. In other respects the respiration is natural, abdominal, without muscular effort or agitation of the alæ nasi. Its extreme rapidity gives it the same appearance as that of a dog which has just ceased running; and this state is exactly expressed by the term *panting* respiration.

"A disturbed state of the respiratory movements occurs only at a more advanced stage of the disease. Then we observe the anxiety of countenance, and the action of the inspiratory muscles. The nostrils are expanded* and the mouth gaping. When the dyspnœa is extreme the lips are contracted, the commissures being drawn downwards and outwards. This is a very fatal sign. Respiration is less frequent than in the former case, and its rhythm is inverted. It commences by a sudden and strong, moaning and jerking expiration, which is followed by a passive inspiration. Each expiration is accompanied by a lateral contraction of the base of the thorax, an enormous protrusion of the belly, and a depression of the subclavicular and sternal regions. I give to the *ensemble* of these phenomena the name of *expiratory respiration,* and if the reader will make a sudden expiratory effort, letting it be immediately followed by an inspiration, he will understand very well what I mean."

The *pulse* is ordinarily of proportionate frequency to the respiration, but

---

* MM. Rilliet and Barthez observe the dilatation of the alæ as most remarkable during the first days of the disease.

in some cases after three or four days this relation ceases to exist. At no age has it been observed less than 120 from the first to the sixth or seventh day, while in the very young it sometimes reaches from 140 to 180. M. Trousseau has counted it 220. Generally it diminishes after the sixth or seventh day, sometimes rapidly so, but in cases about to terminate fatally, the rapidity is resumed. In *cachectic* pneumonia it is usually small and slow.

After describing the state of the *nervous system* in ordinary cases, which is just that which attends the other acute affections of children, MM. B. and R. make a remark respecting some rarer cases which may be useful.

"In these the disease *commences* with violent convulsions, which are often repeated and followed by loss of sensibility. These symptoms then disappear, leaving only a considerable acceleration of respiration and pulse, which should always make us suspect a lesion of the lung; for when the above symptoms result from a phlegmasia of the nervous centres, *the respiration is in general lessened in frequency.* It is exclusively in pneumonia of the apex we have observed these phenomena. Cerebral symptoms are observed in *secondary* but not in *cachectic* pneumonia."

*Forms of the Disease.*—Of these there are three. (1). *Primary* or *Idiopathic Pneumonia.* Gerhard and Rufz formerly stated this was never found in children under five years of age. This Rilliet and Barthez denied in 1838, and subsequent observation has confirmed their statement. (2). *Secondary Pneumonia,* occurring during the course of, or in the convalescence of other diseases, as rubeola and variola. (3). *Cachectic Pneumonia* occurring during the course of chronic disease, or in very debilitated subjects. This is peculiar to young children, and is found especially in the course of chronic enteritis, or at a distant period after the disappearance of the exanthemata. By reason of the absence of active symptoms, such as cough, pain, fever, &c. this disease would seldom be detected but for auscultation, which is of the less consequence, as it is the general state of the system that especially requires attention. Of 245 cases, the authors found pneumonia to be *primary* only in 58, of which number only 24 were less than five years of age. Of the *secondary acute* form there were 102 below and 36 above that age; and of the *cachectic* form, 45 below and 4 above it.

There are several circumstances worthy of note dependent upon the prior state of health, age, and sex of the child.

"The *state of prior health* exerts a marked effect upon the anatomical form and seat of the disease. Thus, at whatever age it occurs, *primary* pneumonia is usually *lobar.* It was so in 55 times out of the 58 cases. The reverse does not however exactly hold good, for of the 187 *secondary* pneumoniæ, 29 were also *lobar,* the 158 being lobular. But still the influence of the former disease exists, and lobar inflammation almost exclusively manifests itself in the course of an acute disease, and very rarely in cachectic subjects. Of 28 secondary lobar pneumonia this form only occurred 4 times in debilitated subjects.

"*Age* exerts a great influence. Thus, of 245 patients, 172 had not reached their fifth year. It has been too generally stated that *lobular* inflammation is peculiar to children from two to six years, although it is doubtless far more frequent then than at a more advanced period. Of 203 autopsies, lobular inflammation was discovered 160 times in children from one to six years of age, and but 43 times in those aged from six to fifteen.

"The influence of *sex* is also multiple. Pneumonia at any age is more frequent in boys than girls. Even allowing for the fact of our having observed a greater number of male than female patients, the preponderance is marked. Of 245 pneumoniæ, 150 occurred in boys and 90 in girls. This predominance of the masculine sex is also more remarkable in lobar than in lobular pneumonia, and in proportion as the child's health was robust prior to the attack."

M. Bouchut, referring to the statement of Valleix, that nearly all the children who die at the Foundling Hospital have hepatized lungs, states that, at his own hospital, of 101 deaths occurring in 1842, but 28 arose from pneumonia. He admits the disease may manifest itself idiopathically, although in the great majority of cases it is secondary. He has observed it especially in Winter, and as often in girls as in boys.

*Prognosis.*—This disease, which is represented by Valleix as nearly uniformly fatal in new-born infants (viz. 127 out of 128 cases), is less so in older children. M. Bouchut found in 55 cases, aged from some days to two years, 33 deaths; and M. Barrier found in 61 cases, aged from 2 to 15 years, 48 deaths. MM. Rilliet and Barthez observe that *primary*, uncomplicated pneumonia is almost always cured in children; but the disease is very grave if it becomes complicated with cerebral or intestinal affections, &c. A second pneumonia may prove fatal in consequence of the deterioration of health brought about during the cure of the first. *Secondary* pneumonia is a much more fatal disease, whose consideration they defer until they treat of the diseases, as measles, &c., during the course of which it generally presents itself. *Cachectic* pneumonia is of all the most fatal.

The symptoms menacing an unfavourable termination, are convulsions occurring at the commencement, notable smallness of the pulse whether in the early or later stage, excessive rapidity of respiration, persistence of bronchial respiration in the very young, slowness of resolution of the inflammation, abundant diarrhœa, and long continuance of cerebral systems. Great emaciation, marked change of countenance, excessive irritability, and a yellowish tinge of the skin are other signs of bad import. Upon this part of the subject M. Bouchut observes:

"There are some symptoms whose importance in the prognosis has attracted the attention of M. Trousseau, who is very skilful in estimating these manifestations. *The swelling of the veins of the hand* coincides with the difficulty of respiration, and is, according to this physician, a very bad sign in pneumonia, proving that the obstacle to hæmatosis, from the extent of the changes in the lungs, is very considerable. It is the same with the *tears*, which manifestation of suffering of the healthy child ceases, when it becomes affected with serious illness. It cries, but there is no secretion unless an evident amelioration in its state has taken place."

*Effect of Dorsal Decubitus.*—MM. Rilliet and Barthez, alluding to the various anti-hygienic predisposing causes, as crowded wards, foul air, bad living, &c. thus speak of the effect of decubitus.

"We possess observations of cases in which the only cause which could explain the secondary pneumonia was the state of inaction in which young children are left in the crowded wards of hospitals. At this age, weakness and the difficulty of expectoration favour the stasis of fluids in the most decumbent parts, and their prolonged sojourn determines inflammation of the bronchi and lung. We say determines, because the anatomical characters of infantile pneumonia have not in most cases appeared to us analagous to hypostatic pneumonia, and we cannot agree to explain only in this mechanical manner lobar and lobular hepatization. We are at issue with M. Gerhard, who believes that the pneumonia of children, from two to five years of age, presents an exact analogy to sanguinary congestions resulting from mechanical obstacles to the pulmonary circulation. It seems to us to deny that pneumonia of young children possesses the true characters of inflammation is to oppose all evidence. The rapid progress, formidable re-action, and obvious changes of structure, sufficiently characterize the disease."

*Treatment.*—MM. R. and B. believe that the cases in which *blood-letting* is advisable have not been sufficiently discriminated, the local lesion having been too exclusively kept in view. Leeches or general bleeding in the *primary* disease are essential, but in the *secondary* form their use is the exception—being then confined to cases in which the child is not very young, the primary disease has not long existed, and the debility is not too considerable. *Tartar-emetic*, in contra-stimulant doses, exerts a marked effect in diminishing the rapidity of pulse and respiration, but when it is given early and alone this is not permanent. The best treatment is the combination of bleeding with it, in all cases except those in which great debility prevails, but especially in the idiopathic form. In cases not admitting of such decisive treatment the *white oxide of antimony* in very large doses is an useful remedy. Small doses of *Kermes* are sometimes beneficial. The Germans prefer giving antimony after bleeding in *emetic* doses, and the authors approve of this when there is considerable accompanying bronchitis. In persistent hepatization too, the Germans are very partial to calomel and digitalis, but our authors prefer the contra-stimuli. *Tonics* are required after the resolution of the inflammation, and much harm results, especially in the secondary form of the disease, from a too long persistence in antiphlogistics. The authors do not approve of *blisters* and other exutories, believing they often produce an injurious re-action, and in the emaciated and feeble state of the body in secondary pneumonia may do much harm. They use them when bronchitis is also considerable.

We agree with M. Bouchut in preferring ipecac. to the tartar emetic, on account of the occasional prostrating effects of the latter. M. Valleix, however, employs it conjointly with bleeding. M. Bouchut does not hesitate bleeding young infants when they manifest sufficient power of re-action ; and, in those cases in which re-action is nearly or quite absent, he recommends quinine. He has not the same fears of blistering as the authors above quoted have ; and states that he has frequently seen an emetic and a very large blister, occupying the whole of the posterior part of the thorax, cut short the disease in its earliest stage.

### *Emphysema of the Lungs.*

This affection is noticed here because, in the great majority of instances, it is a consequence of bronchitis or pneumonia, although it may be also produced by other causes inducing great acceleration of respiration, with obstruction to the penetration of the air. The emphysema is usually seated at some distance from the inflamed portion, thus, *e. g.* the latter is found chiefly in the inferior, and the emphysema in the superior lobes. *Chronic* emphysema, so frequent in the adult, is *not found in the child,* except indeed when it results from the compression of the lung in rickets. *Vesicular* emphysema is infinitely more frequent than *interlobular;* for Rilliet and Barthez found in 134 cases 126 were vesicular, and 19 interlobular. In 11 the two lesions co-existed; and in 115 the vesicular, and 8 the interlobular form existed alone. Bouchut, on the contrary, speaking of younger children, says, "we very frequently meet with interlobular emphysema, hardly ever with vesicular." The lungs are more or less distended, accordingly as some lobules, a lobe, or an entire lung, are affected. The vesicles, of the size of pin's heads, and never so large as hemp-seed, are seen by the naked eye, and upon dividing them their walls are not found thickened. The affection is usually only partial, occupying especially the anterior border and apex of the lung. The physical signs are different from those offered by the adult, for while in the latter the respiratory murmur is feeble and the parietes are sonorous and dilated, in the child, the murmur is exaggerated, and the chest not more sonorous than in health.

### *Pleurisy.*

Idiopathic pleurisy was formerly believed never to occur in children, but the investigations of the authors and others, prove this opinion to be erroneous. In very young infants it is a rare disease, for they have only met with three cases below five years of age, and M. Bouchut relates but two others. The secondary disease is not rare in these, for the last-mentioned author found in 68 autopsies, marks of pleurisy in 23. It is usually, however, a complication that adds little to the gravity of the original disease. Pleurisy is much oftener unilateral than double, the right side being that most frequently affected, except when it is complicated with secondary pneumonia, when the left predominates. False membranes are the most usual inflammatory product, but they are usually soft, thin, and loose, and do not form firm adhesions, as in adults. They are found at the lower part of the cavity. The quantity of serum effused is seldom considerable.

Rilliet and Barthez mention *bronchial respiration* as a very early symptom of the disease; being audible on the second or third day. Its sound is peculiar and metallic, and differing from that in pneumonia. It is usually heard on one side only, and especially in the vicinity of the inferior angle of the scapula and intra-scapular spaces. It sometimes disappears at the end of two or three days, and may then re-appear or not. They explain the frequent occurrence of this sound by (1) the small capacity of the chest in the child, (2) the greater number of inspirations, (3) the small quantity of fluid effused. Mostly it is accompanied by *ægophony.* Absence or feebleness of respiratory murmur may co-exist, but is usually found in chronic cases or debilitated subjects. When the pleurisy supervenes on pneumonia, occasionally a complete absence of respiratory murmur replaces the bronchial respiration; but in most cases the latter becomes augmented in intensity, and even cavernous, so as to lead to the belief of the existence of a cavity—the voice at the same time resounding with such force as literally to pierce the ear. M. Bouchut notices as a very marked sign *the absence of vibration*, upon the application of the hand to the chest during the respiratory movements or cries of the child. In *pneumonia*, on the contrary, this vibratory movement is much *augmented*. The *dyspnœa* is not so urgent in the primary form as it is in pneumonia (the alæ nasi not being dilated at so early a period), and the respiration frequently becomes almost normal again about the 4th or 6th day. In pleurisy which is secondary to a pneumonia, the oppression of breathing is sudden and extreme.

*Prognosis.*—Simple primary pleurisy is usually cured, and secondary pleurisy is a less fatal disease than secondary pneumonia. Pleuro-pneumonia is a very dangerous disease, and *cachectic* pleurisy is almost constantly fatal. Of the 60 cases adduced, 21 were simple and primary, and all recovered; 12 secondary, of whom 3 died; 5 primary pleuro-pneumoniæ, of whom 2 died; 10 secondary pleuro-pneumonia, of whom 8 died; 5 chronic pleurisy, of whom 2 died; and 7 cachectic pleurisies, all of whom died. Secondary pleuro-pneumonia is the only form of the disease found most frequently in children less than 5 years of age; while 5 only were found in 21 pleurisies, 44 were found in 61 pleuro-pneumonies.

"*Influence of Prior Diseases.*—Just as bronchitis is one of the most frequent causes of the development of pneumonia, so pneumonia exerts an evident effect in the production of pleurisy. But frequently the inflammation of the pleura consists only in a few false membranes, or the effusion of a little fluid, constituting no serious disease. However, in other cases, the pleurisy becomes as grave a disease as the pneumonia itself. A number of diseases which are considered as predisposing causes of pleurisy, have in themselves no direct influence in this way: but as they are frequently complications of pneumonia, we see in this inflammation the true cause of pleural phlegmasia. Thus, nothing is so rare as to see measles complicated with simple pleurisy; while rheumatism, scarlatina, and Bright's disease, are the affections in the course of which simple pleurisy ordinarily occurs. We must not, however, confound with pleurisy the acute hydrothorax which so frequently complicates the diseases we have mentioned."

*Treatment.*—Leeches for young children, and general *bleeding* for older ones, if had recourse too at an early period, will rarely require repetition in acute pleurisy. In the secondary and chronic forms a small loss of blood may also be required. The side affected should be enveloped in a *cataplasm*, and abundant diaphoretic or diuretic drinks given. Where the disease does not yield to a bleeding, and is of an active type, the administration of *tartar-emetic*, as in pneumonia, is useful. This medicine is of no avail in simple secondary pleurisy, attended with great orthopnœa. In these cases, *calomel*, united with *digitalis*, as employed by the Germans, is of great use: but it is especially in chronic pleurisy that mercurials are indicated. *Diuretics*, as nitrate potass, digitalis, &c. may be given as adjuvants in small doses, in the diluent drinks taken by the child; but in secondary pleurisy, attended with suffocative symptoms, which follows generally eruptive fevers, and proceeds with great rapidity, and mercurials have not proved useful, digitalis or squill should be given in full doses. *Purgatives* are chiefly of use in chronic pleurisy. *Blisters* and other derivatives are not approved of by the authors; but they think advantage is derived in chronic pleurisy from enveloping the side in a large diachylon plaister. *Tonics* are required in the chronic or cachectic forms. The operation of *paracentesis* is spoken of by the authors in a favourable manner, affording as it does more chances of recovery in the child, in whom chronic pleurisy, independent of phthisis, is more frequent than in the adult. They do not, however, cite any facts of their own, but refer to those reported by M. Heyfelder. M. Trousseau has published a paper in the 12th volume of the Annales de la Chirurgie, highly approving of this operation.

### *Pericarditis.*

This is a rare and almost always a secondary affection in children, occurring almost exclusively in children less than six years of age, and generally in the course of rheumatism. The remarks of the authors are founded upon twenty-four observations. The quantity of fluid secreted is usually small, and the density of the false membranes much less than in the adult. When the disease is slight it does not furnish distinct symptoms. It does not seem in itself a very dangerous disease (except where the whole pericardium is affected), death having usually occurred, in the fatal cases, in consequence of the primary affection. The treatment is very similar to that required for pleurisy, digitalis as a means of calming the action of the heart, and mercurials as inducirg absorption, being especially indicated. Dr. Alison, in the 35th volume of the Medical Gazette, relates some interesting cases of pericarditis supervening on scarlatina.

### *Coryza.*

This, which in its simple form is usually a very slight disease, becomes a very severe one in the chronic and pseudo-membranous forms. M. Bouchut describes a chronic syphilitic form as not of rare occurrence, and as best relieved by the iodide of potassium. Pseudo-membranous coryza is a rare disease, and is seldom observed unconnected with the same form of angina. The dense false membranes which are formed, powerfully impede respiration, and the obstruction gives a peculiar resonance to the cough. The disease is usually fatal. If pseudo-membranous affections are not raging epidemically, a few leeches may be applied to the mastoid process. Where depletion is contra-indicated, revulsives to the lower extremities and calomel should be employed. After the use of emollient injections, astringents, as powder of alum and gum āā, or calomel and gum, may be blown into the nostril, or a solution of argent. nitr. may be applied by means of a pencil or glass syringe.

### *Stomatitis.*

This may manifest itself in the *pseudo-membranous* and *ulcerative* forms, and the latter must not be mistaken for gangrene of the mouth, which will be treated of hereafter. The ulcers usually commence on the gums, and the insides of the cheeks are afterwards affected ; they are sometimes very deep, but on feeling with the finger we do not perceive the hard circumscribed engorgement surrounding them, as in gangrene, but a soft flabby sensation. Nor does the external skin present the hot, shining, smooth appearance, as in gangrene. The fætor of the breath is very similar, and in bad cases abundant salivation occurs. The prognosis is much more favourable than in gangrene, a cure usually soon following proper treatment. It occurs especially between the ages of 5 and 10, affecting children of feeble habit or exposed to adverse hygienic circumstances, and those who have long suffered from other diseases. It is endemic in some wards of children's hospitals, and M. Taupin considers it contagious.

Improving the hygienic condition of the child and the use of an acidulated gargle, often suffice for a cure. When the inflammation is very severe leeches are required, and rubbing dry *chloride of lime* into the part with the point of the finger twice a day is the best application, washing the mouth with water a few seconds after using it. When the cicatrization commences, a gargle, formed of 1 part of the chloride, 30 of mucilage, and 15 of syrup, may be used. If the chloride be left off too soon, relapse is sure to occur. M. Bouchut prefers this application, honey 15 to 20 parts, hydrochloric acid 3 to 5 parts. M. Trousseau cauterizes the parts with nitrate of silver or hydrochloric acid, and uses in the intervals a paste of equal parts of honey and borax. A good diet and free exposure to pure air are essential.

M. Bouchut has an interesting chapter upon the *Muguet*, which is not a simple stomatitis. According to M. Gruby, the white deposit in these cases is formed by a cryptogamous vegetable parasite, and is seated externally to the mucous membrane, although the epithelium is separated in removing it. It occurs occasionally as an idiopathic disease in children of bad health, but usually in the course of other diseases, especially entero-colitis, pneumonia, and phthisis. It is not a fatal disease, and the author cannot understand the statements of MM. Valleix and Baron, who represent it as highly so. It must arise from their representing death as occasioned by *muguet*, which really resulted from the primary diseases. Valleix states that 22 out of 24, and Baron that 109 out of 140 died. Of 42 patients, Bouchut found 14 affected with the idiopathic disease, who all recovered, 20 died affected with entero-colitis or pneumonia, and 8 still remained affected with the original diseases and the muguet. The disease is oftener found in infants at the breast, than at any other age. It may be propagated by contact. Various emollient and astringent applications have been recommended; but honey and borax in equal parts is by far the best one.

### *Diphtheritis.*

This, the *pseudo-membranous* form of *pharyngitis* or *angina*, may occur as a primary disease, as well as consecutively to the exanthemata. The deposition of the firm false membranes is characteristic, the mucous membrane beneath being sometimes smooth, at others ecchymosed or ulcerated, especially in the secondary form. The febrile re-action is not ordinarily intense, and the sporadic uncomplicated form is of short duration, while the degree of febrile action in the secondary form depends upon the primary disease. Bretonneau first proved this not to be a gangrenous form of angina as formerly it was believed to be, the error having arisen from putrefying false membranes being mistaken for eschars. Gangrene in its progress and symptoms is quite a different disease, but in some epidemics it may have prevailed coincidentally with the pseudo-membranous angina. Gangrene chiefly attacks debilitated children, while this affection equally attacks the most robust. The *prognosis* is usually very favorable in the sporadic form, and is not unfavorable in the epidemic form, when there is not a great tendency in the disease to spread to the air-passages or to the skin, producing croup in the one case and abundant suppuration in the other. In the secondary form the prognosis is much less favourable. The disease is very frequently epidemic in France and Switzerland, and the authors believe it to be contagious. The *treatment* must at first be antiphlogistic proportionate to the character of the epidemic; but *local* applications are most to be relied upon. These are hydrochloric acid, nitrate of silver, alum, or chloride of lime, in different cases. The caustic should be applied in its strong state once in the twenty-four hours, and afterwards oftener, but diluted, taking care always to go beyond the margin of the part affected. Emetics are useful adjuncts, and *mercury* should be employed if the air-passages are implicated, while, in the feeble, tonics are required.

### *Croup.*

We need not transcribe the author's description of the well-known anatomical characters of the disease; but it may be useful to remember that, of 120 cases collected by M. Hussenot, the false membranes extended to the bronchi in but 42, and not beyond the trachea in 78. Even when the bronchi do not contain membranes they are usually inflamed, reddened, softened, and more or less filled with mucus or pus. In $\frac{5}{6}$ of the cases observed there was lobular pneumonia, and emphysema existed in the majority.

After detailing the *symptoms* of croup, MM. R. and B. thus remark upon their want of correspondence with the lesions.

"Let us enquire whether the extent of the lesions is proportionate to the intensity of the symptoms? In a great number of cases it evidently is, while in many others it is far from being so; and if large cylindrical pseudo-membranes obstructing in a great measure the larynx, trachea, and bronchi, are accompanied by formidable symptoms, symptoms no less severe exist in the case where the lesion is far more limited, or where it is but rudimentary. Numerous examples are found in authors where the most frightful symptoms of croup have existed, and yet the autopsy has only shewn a few shreds of pseudo-membranes in the larynx, and sometimes even only in the trachea. Moreover, is there not a disease which offers so great a similitude to croup as to have been confounded with it by most authors, and in which no sort of change is found after death in the laryngeal mucous membrane, or only a slight inflammation without tumefaction capable of obstructing the air-passages? Then, do not cases of secondary croup prove that false membranes, or inflammation of the mucous membrane, will neither account for all the phenomena. * * * * * * * * * * "The symptoms of croup seem to us then to depend simultaneously or solely upon the presence of false membranes, of inflammation of the mucous membrane, and of spasmodic contraction of the larynx. This last may alone produce suffocative paroxysms and acute laryngeal wheezing (heard during inspiration), and that when no false membrane covers the *cordæ vocales*. We cannot attribute much influence to inflammation of the mucous membrane, since it is accompanied by so little swelling in croup. We do not believe in a sudden swelling of the glottis, because the wheezing and suffocation come on without obvious cause, or under the influence of some moral cause, as passion,&c.—the same causes in fact that determine the paroxysms of pertussis. The presence of the false membrane explains a part of the symptoms of croup. But the existence of these symptoms in the absence of the pseudo-membranous concretions renders another explanation necessary. The temporary swelling of the glottis, which is nowise proved, cannot account for the phenomena, *while spasm of the larynx* explains them perfectly, and its existence is justified by the analogy of other nervous diseases."

*Tracheotomy in Croup*.—A valuable paper from the pen of Professor Trousseau is introduced into both these works. The opinion of observers in this country is not much in favour of the operation, and it is well to see what so able an advocate has to say in its favour. M. Trousseau observes that, as the operation in itself is not dangerous, the ill success which has often attended its performance in laryngitis and croup has much arisen from its being delayed until the phenomena and consequences of asphyxia have shewn themselves. Tracheotomy is to be preferred to laryngo-tracheotomy. The dangers of the operation have been much exaggerated, for in 121 operations, M. Trousseau has had but one fatal accident during their performance. An adult died the instant an incision was made into the skin.

"It has doubtless happened that I have met with arterial anomalies: but as I always make it a duty to operate very slowly, and never to make a stroke with the knife without being certainly directed by the finger and the eye, I am persuaded I should avoid the left carotid, if it should be given off from the innominata and cross the upper part of the trachea. As to the trunk of the innominata, I have had it several times under the edge of the bistoury, but in bending my incision a little to the left, and separating the tissues with the finger and with the *erigne*, I have completed without fear or accident operations apparently so dangerous. Those surgeons who pride themselves upon performing the operation with a marvellous rapidity, and plunge the bistoury boldly into the trachea to divide it from below upwards as soon as they have finished the incision of the skin, will deplore this imprudent and useless celerity, when they find under the edge of the knife vessels which it is so easy to avoid when it is more the object to operate safely than quickly."

One important reason for preferring the trachea as the site of the operation is, that the necrosis of the cartilages, which is often excited by the presence of the canula, is of less consequence there than it would be in the larynx. The thyroidean and other large veins should be avoided as much as possible during the operation, and should never be tied if divided. When blood has been accidentally introduced into the trachea, M. Trousseau has never found ill effects result, if the lips of the wound have been held open, or the canula at once introduced. The canula becomes a valuable means of introducing topical applications to the disordered mucous membrane. Thus a strong solution of nitrate of silver (1 part to 5 of water) may be applied by means of a little mop, at first three or four times and afterwards once daily, a weaker solution being occasionally injected as well.

Water injected in small quantities frequently relieves irritation and obstruction, and assists the expulsion of false membranes, &c. M. Trousseau thus concludes his paper.

" With these means of treatment, which are carefully employed by M. Bretonneau and myself, the success has not been very brilliant. Yet in twenty operations M. Bretonneau has saved six children, and in 112 I have saved 27. M. Leclerc, who adopts this operation, has saved the two children he has operated upon. M. Velpeau has cured two out of ten, and M. Petel, three out of six. Thus of 150 operations success has attended in 39. I regret I cannot give the results obtained by many other of our *confreres*, who have adopted the therapeutical means recommended by M. Bretonneau and myself. The comparison of results would have been interesting, but the materials are wanting. What, however, we know very well is, that in the practice of MM. Gerdy, Robert, Guersant, &c. &c. there are living in Paris nearly fifteen children who have had tracheotomy performed in the last stage of croup, and in whom our subsequent plan of treatment has not been followed.

" I believe I may add some propositions in relation to the prognosis which may prove of some interest.

" 1. If the disease began several days ago, and the croup consequently makes slow progress, the children, whatever be the extent of the false membranes, will either be cured, or will live at least for several days. 2. But if its progress has been very rapid, although it should prove at the time of the operation that the false membranes did not extend beyond the larynx, the children die very quickly. 3. If, prior to the operation, the false membranes invade the nostrils, if they cover the surface of blisters, if the child is pale, and is a little bloated without having been bled or taken mercury, or if it has been much bled, the operation offers but little chance. 4. If, before the operation, the pulse is moderately frequent, and continues quiet after it, there is hope. 5. Rapid respiration immediately after the operation, with little or no cough, is a bad sign. 6. More boys than girls are cured. 7. Children less than two, and more than six years of age, are seldom cured. 8. All things being equal, the danger is great in proportion to the extent of the false membranes. 9. If the child is subject to chronic catarrhs, and if it had taken cold some time before the croup seizing it, the chance of success is the greater. 10. When all seems going on well, rapid respiration is a bad sign. 11. The more rapid and severe the inflammatory action which seizes the wound, the more probable the cure. The sudden sinking in of the wound is a mortal sign. 12. There is nothing to fear as long as the respiration is normal, or noise is only produced by the displacement of mucosities; but if it becomes *serratic* (i. e. noise like that made in sawing stone), death is certain. 13. We must not despair even if a pneumonia or pleurisy supervenes. 14. Restlessness and sleeplessness are bad signs. 15. If the wound becomes covered with false membranes; if, on the removal of the canula, it does not contract promptly; if, when nearly cicatrized, it becomes widely re-opened, there is danger. 16. The sooner we are enabled, in consequence of the freedom of respiration through the larynx, to remove the canula, the more certain and rapid is the cure. 17. The operation does not succeed in croup supervening upon measles, scarlatina, small-pox, or pertussis. 18. If, on the third day after the operation, the expectoration becomes mucous and catarrhal, the children are cured; if it is absent, serous, or resembles little pieces of half-dried gum, they die. 19. If there is considerable re-action upon the injection of water or nitrate of silver, we must not despair, however bad the other symptoms may be. 20. Children seized with convulsions die; and convulsions are more likely to occur as the child is young, and has lost much blood before or during the operation. 21. When, after the 10th day, drinks pass almost entirely from the pharynx into the larynx and trachea, although they may be easily rejected, the children generally die."

*False Croup, or Spasmodic Laryngitis.*—This affection is not considered by MM. R. and B. as purely spasmodic, but as compounded of a spasmodic and inflammatory element. They observe it has been recognized in France long after its admission as a separate disease in England; and they attribute the success of many vaunted remedies for croup, and accounts of relapse, to this form being mistaken for the true one. The diagnosis is often very difficult, and in some cases impossible, in the early stages. The following signs are enumerated by Wichmann.

"False croup—1, Comes on suddenly, the first attack almost always manifesting itself at night. 2. It is always sporadic. 3. When the cough exists it is dry, without any expectoration. 4. Pain is absent, but there is a sense of constriction of the thorax. 5. The voice is raucous or hollow. 6. There is no fever. 7. The symptoms are alternated with intervals during which the children seem quite well. *True Croup*—1. Commences slowly and by degrees, the first attack generally coming on in the day. 2. It is generally epidemic, rarely sporadic. 3. Layers of puriform matter or membranous concretions are expelled by coughing or vomiting. 4. Pain exists in the air-passages, and slight tumefaction may be felt though not seen. 5. The voice possesses a sound quite peculiar. 6. There is fever. 7. There are not evident intermissions."

M. Bouchut observes—

"The progress of this affection is essentially different from that of true croup. We find a sudden increase of frightful symptoms of suffocation, which cease to re-appear with less and less intensity, until they disappear entirely. The intermissions are well-marked, during which the general health remains good. Croup is characterized by its increasing gravity. The attacks, slight at first, become more and more violent until they threaten or cause death from asphyxia. In the intervals the children are suffering under a horrible difficulty of breathing, the colour of the face indicating the magnitude of the obstacle to free respiration. So, too, on examining the back of the mouth, we may often detect the existence of characteristic false membranes."

Still the practitioner is often left in doubt, and feels himself compelled, on account of the fearful rapidity of progress of the true disease, to treat the case more energetically than he would do if he could assure himself it was an example of false croup. Even the spasmodic form may prove fatal if the suffocative paroxysms are frequent and intense. Pretty free, though not excessive depletion, revulsives to the lower extremities, emetics and purgatives, antispasmodics, as musk and assafœtida, and narcotics, as belladonna, are the means recommended by the authors. They notice also Dr. Lehman's plan of applying very hot sponges (hot salt will do as well) incessantly until diaphoresis is excited. This simple means should always be had recourse to instantly in any paroxysm of a croupy character, and it will often render farther measures unnecessary.

### *Gastro-Enteritis.*

MM. Rilliet and Barthez give, prior to describing the individual affections, a good general view of the physiological condition, cadaveric changes, and pathological anatomy of the gastro-intestinal mucous membrane.

This our space forbids quoting. Among other facts they observe that mere redness or ramollissement, taken alone, may result from cadaveric changes, but when seen together, they usually are the consequences of inflammation. In cadaveric injection the vessels are larger and intercommunicate much more freely, while the colour is a dull violet rather than a brilliant red. In non-inflammatory ramollissement the mucous membrane is generally quite pultaceous, or, when it is less so, of a milky whiteness. In inflammation, too, the consistence may be pappy, but, in most cases, small portions of the softened membrane may be raised entire. If *thickening* is added to the above appearances, inflammation has certainly existed. Its degree is not always appreciable, slight projections of different points of the mucous membrane giving it the appearance of enlarged villi. The polish of the membrane is replaced by a peculiar shagreen appearance. When chronic, the submucous tissue becomes hypertrophied and indurated; but a contracted condition of portions of the canal may simulate this appearance. *Ulceration,* even independently of typhoid and tuberculous disease, is not uncommon in children. The follicular ulcers, commencing in small serpiginous lines, eventually occupy a vast extent of membrane. They differ from tuberculous ulcers in not penetrating the submucous tissue, and they are sometimes covered by a false membrane. In a few cases, in all of which tartar-emetic had been given, a kind of *pustular* inflammation was found to be developed.

1. " *Gastritis and Ramollissement of the Stomach.*—The time is not very distant when gastritis was the pivot upon which all pathology turned. The observation of facts not having justified the conceptions of genius, inflammation of the stomach has become regarded in our day as one of the most rare of diseases. In this there is some exaggeration. Perhaps the re-action has gone too far in attributing to the stomach a kind of immunity against inflammation, especially that which results from irritants directly applied to it. However this opinion may be disputed as regards the adult, it cannot be in respect to the infant. But, while stating that gastritis is more frequent and more easily produced in the young than is generally admitted, we have no difficulty in allowing it does not deserve an important place in the nosology of childhood. When primary it is almost always a slight affection, and when secondary it is but an epiphenomenon of an already dangerous disease, or the result of an active use of medical means. Lastly, it is often latent, escaping all investigation. We have always found it in an acute form. What we say of gastritis we may repeat of ramollissement. We find ourselves at variance here with a considerable number of practitioners, who consider this as one of the most grave affections of childhood. Our observations have in fact only led us to look upon it as a secondary lesion, and never as a primary disease, influencing the entire organism, revealing itself by special symptoms, and observing a regular progress. The *age* of the subjects who have formed the objects of our study is doubtless the cause of this difference of opinion."

M. Bouchut, alluding to young infants, denies the *gelatiniform ramollissement* of the mucous membrane of the stomach, upon which so much was written a few years since, to be a separate disease at all. He says it is never found alone, but always as a consequence of inflammation of the intestinal canal, during which abundant acid secretions take place, which act upon the gastric membrane purely in a chemical manner.

MM. R. and B. reserve all remarks upon tubercular diseases of the abdomen, and in 61 autopsies of non-tuberculous cases, found 21 examples of erythematous, four pseudo-membranous, one pustular, six ulcerative gastritis. In two cases eschars had formed, and in 27 there were various degrees of ramollissement. *Vomiting* is a chief symptom, but it is not always present, and the statement of authors that ramollissement is always attended with constant, severe, and abundant vomiting is erroneous. The *thirst* is ardent and excessive, but the *tongue* is rarely red or dry, but mostly white and moist. *Diarrhœa* is usually present. In more than two-thirds of the cases cited the disease was induced by the use of tartar-emetic mixture or kermes mineral for the relief of the original disease; but these medicines must not be too indiscriminately proscribed, as in great numbers of children they produce no ill effect whatever. Strong and robust children are oftenest the subjects of ordinary gastritis, and the very young, feeble, and cachectic of ramollissement. Meningitis is the disease in the course of which gastritis usually shows itself, then the eruptive fevers and thoracic or abdominal phlegmasiæ. If the child is strong enough, cupping or leeching the epigastrium is requisite. It should be covered with a cataplasm, and emollient drinks given. Icy drinks or fragments of ice allay the thirst. Opiates are useful. Total abstinence at first, and afterwards small portions of milk.

2. *Inflammation and Ramollissement of the Intestines.*—Inflammation and ramollissement, especially of the large intestine, is one of the most common and destructive lesions of childhood. Including tuberculous cases, of every two children who die, in one will there be found lesion of the large intestine, and indeed it is rare for a child to die between two and five without manifesting such. M. Bouchut represents the disease however as almost special to children less than two years of age, but then he excludes tuberculous cases, as tubercles are not found in the intestines in sucking children. Although ulcers may be found in the whole course of the intestine, they are so especially in the sigmoid flexure and rectum. The following are the relative numbers of the non-tuberculous lesions observed. Erythematous, pseudo-membranous, or ulcerative inflammations of the small intestines 45, of the large 113; follicular inflammation of the small 90, of the large 64; ramollissement of the small 28, of the large 35. Lesions of the stomach may sometimes exist alone, but various parts of the intestines are usually simultaneously affected.

*Diarrhœa,* in abundant quantities, is a common *symptom,* although it is absent in about one case in twelve; and as a result of about 300 autopsies the authors observe that, of twelve children affected with considerable diarrhœa, in one only will the intestinal canal, on post-mortem examination, be found quite healthy. M. Bouchut justly objects to referring all diarrhœas, as Rilliet and Barthez do, to the presence of a phlegmasia, and describes two forms, the catarrhal or spasmodic, and the enteritic. He makes the following observations upon the connexion of *diarrhœa* with the process of *dentition.*

"It may not be uninteresting to state this with more precision than has been done hitherto. In the cases of 110 infants we have enquired what has been the condition of the alimentary canal during the first dentition. A small number (26) have suffered no indisposition. In 38 there had been restlessness, colic, and temporary diarrhœa, not severe enough to excite the fears of the parents. In 46 there was copious diarrhœa. In 19 of these it appeared at the same time as the irritation of the gums, and ceased on the cutting of the teeth. In 28 cases, where the teething was very difficult, the purging persisted, and took on all the characters of enteritic diarrhœa.

"It is difficult to determine satisfactorily the relation which exists between the process of dentition and the irritation of the intestinal canal. According to some, the pain occasioned by the irritation of the gums occasions a nervous irritation, which augments the peristaltic action, and impedes the assimilation of food. The diarrhœa being then considered a nervous and sympathetic affection, others regard it as resulting from an extension of the inflammatory condition of the mucous membrane of the mouth to other portions of the canal. These opinions may be just, but they need not be adopted to the exclusion of each other. Diarrhœa dependent upon dentition seems at least at first to be a sympathetic affection; and it is only when it has become long-established that it takes on the characters of an inflammatory flux."

Inflammation of the intestinal mucous membrane (entero-colitis) is considered by Rilliet and Barthez under the following forms. (1). *The acute primary normal form,* which is usually but a trifling disease. (2). The *acute secondary normal* form, which is the most frequent, occurring in the course of measles, scarlatina, &c. Its production is stated by the authors to occasionally arise from the injudicious use of *purgatives* during the primary disease. The lesions discovered after death do not always correspond with the symptoms observed during life. (3). The *acute typhoid form* is a rare disease intermediate between normal enteritis and typhoid fever, and distinguished with difficulty from the latter. (4). The *dysenteric form* is also rare, only seven cases having been observed, all of which exhibited very extensive ulcerations. (5). The *cachectic or chronic* is a very fatal form, especially in hospital practice, and produces a degree of feebleness and wasting seen in no other disease besides tubercle. Its origin can usually be traced back to the period of dentition, weaning, or some sudden change of diet. The authors agree with M. Trousseau as to the *danger of neglecting diarrhœa* at the *period of dentition.* They consider the fears of arresting it are groundless, and that it should not be allowed to go on unchecked more than four or five days. In this chronic enteritis astringent enemata, as tannin or argent. nitr., are indicated, and M. Trousseau recommends the latter by the mouth, also ℞. Nitr. Arg. gr. $\frac{1}{5}$, Aq. Dest. Syrup. āā ℥vj. divide into 8 or 10 doses. Tonics, such as calumba and cascarilla, or if the diarrhœa is obstinate, iron in the form of pernitrate or in chocolate are required. In mild cases, saccharate of lime, subnitrate of bismuth, or a little bicarb. sodæ, added to the milk, may be given as antacids. Hygienic and dietetic rules are of the last importance.

### *Peritonitis.*

Acute peritonitis is alone treated of, leaving the chronic until tuberculous diseases are considered. It is a less frequent disease than pericarditis, and much less so than pleurisy. The chief symptoms are marked tension and swelling of the belly, often offering resistance to pressure. When the disease is partial, there is to appearance a painful tumour formed. The pulse is very small, ranging from 120 to 140. The countenance is pale, anxious, and shrunken. M. Bouchut observed in two cases a peculiarity in the respiration, which was remarkably short and incomplete, while every 8th or 10th inspiration was a very deep one, as if for the purpose of making up the deficiency of the others. Of the twelve cases referred to by R. and B. four were primary and eight secondary. It is a very fatal disease.

### *Hepatitis.*

This is a rare and mild disease in children. The symptoms are jaundice, tumefaction, and pain. *Jaundice* independently of this affection is not found in children (except new-born infants, in whom too the liver and other abdominal organs are much engorged) independently of inflammation. The authors cannot allow that this is the dangerous disease it has been represented by Burns and Henke to occasionally be.

### *Nephritis.*

The authors observe that *albuminous* is not very easily distinguished from ordinary nephritis in the child, inasmuch as there is an absence of Bright's granulations, even when anasarca and albuminous urine had been observed long prior to death. The yellow colour of the third stage of ordinary nephritis nearly approximates to that observed in the albuminous form. In 23 out of 48 cases of nephritis the urine was found albuminous, and in 16 was not examined. There is not the same febrile action as in the simple nephritis of the adult; but in the albuminous form there is anasarca, which is often complicated with effusions into cavities, especially the pleura. The proportion of chronic to acute cases is much less than in the adult. One third of the whole cases occurred during the desquamation of scarlatina, exposure to cold seeming the exciting cause. It is more rare after measles. Children above five years are most liable. The *treatment* consists, in acute cases, in depletion, warm baths and purgatives. In the more chronic form, sudorifics and diaphoretics. Frequent vapour baths, prolonged to the induction of profuse sweating, have been useful in all forms. Digitalis, or nitrate of potass, may also be given, and cataplasms applied to the lumbar regions.

### *Inflammatory Affections of the Brain and Spinal Marrow.*

The authors do not allow that affections of the brain constitute so large a proportion of the diseases of children as they are generally stated to do.

1. *Simple Meningitis.*—Meningitis, when unconnected with tubercle, is a rare disease. Only six cases are alluded to. The chief diagnostic signs are intense headache, abundant vomiting, and high fever, rapidly followed by delirium, excessive anxiety and collapse. Active antiphlogistics can alone save the child.

2. *Affections of the Cerebral Sinuses.*—Five cases are added to the twelve already published by M. Tonnelé. The superior longitudinal sinus is that usually affected. It is found more or less filled and distended with firm coagula, which sometimes too extend into the cerebral veins. Pus is also occasionally found in the centre of the coagulum or mixed with blood. The lining membrane of the sinus usually retains its natural appearance, but the cellular coat is much thickened. These concretions may determine hæmorrhages or effusions, but are denoted by no special symptoms. They are only found in the feeble or cachectic.

3. *Cerebral Congestion.*—Great difference of opinion prevails as to whether this is other than a secondary phenomenon, or even a mere post-mortem appearance. It has been found in the absence of all, and after quite opposite symptoms, and is certainly a less important sign of disease in the child than in the adult.

4. *Ramollissement* of the Brain, so frequent in the aged, is rarely if ever seen in the child as a disease sui generis. It has been found in children who have succumbed in various diseases, and in whose ventricles serum has existed, as also in the vicinity of old cerebral lesions. A case of idiopathic ramollissement is however related, and an example of it supervening upon encephalitis.

5. *Hypertrophy and Induration of the Brain.*—A general induration or increase of consistence is sometimes found without, or at others with, augmentation of bulk. The authors allude to the cases described by Laennec, Papavoine (in lead poisoning) and others, but do not state their own experience.

6. *Spinal Meningitis.*—Only one example is adduced, and, as in nearly all the cases related by other authors, it was complicated with tuberculous disease of the brain.

7. *Ramollissement of the Spinal Cord* is a more frequent lesion, but its existence may be simulated in any subject if the spinal cord is contused in opening the canal. It may manifest itself under three forms : as an acute disease with symptoms hardly to be distinguished from tetanus ; an acute disease with symptoms resembling chorea, and as a chronic disease attended with more or less complete paralysis.

### *Articular Rheumatism.*

This is a rare affection in the child, and only occurs in the acute form. Ten cases, all occurring between the ages of 12 and 14, were met with by the authors. The disease is rendered much more severe when it becomes complicated with pericarditis or pleurisy, but even then recovery usually takes place. Leeches and oily embrocations in mild cases, and active depletion in severe ones, are indicated.

### *Cutaneous Inflammations.*

As the authors defer the description of the exanthemata, there is little here to detain us. We may extract a passage upon an interesting subject, namely, the *effects produced upon the internal organs by the repulsion of cutaneous eruptions*, especially those of the scalp.

"We commence by observing that many of the facts cited as proofs of the danger of repulsion will not bear analysis. Generally no exact account is given of the state of the general health prior to the disappearance of the eruption, so that it is difficult to determine whether the production of the internal affection is the effect or the cause of the disappearance of the external disease. On the other hand, we have been able to assure ourselves, from observing a great number of cases, that in reality the internal phlegmasia is almost always anterior to the other. We feel obliged then to restrict the influence which is generally accorded to the retrocession of dartres and exanthemata, and which, from age to age, from school to school, has been constituted a corner-stone of the pathological edifice. We do not deny the existence of the coincidence of serious accidents, and the sudden disappearance of cutaneous disease, but we maintain that this is much less frequent than is generally supposed, and may be sometimes explained by another theory than that of retrocession.

"Experience teaches us that when the inflammation occupies but a limited surface, we may hasten its disappearance by appropriate means. On the other hand, when it is very extensive, the rapid fall of the whole of the crusts which cover the scalp, and the contact of the air with a vast suppurating surface, may give rise to dangerous results, as the production of cerebral disease. As we have said, other explanations besides that of retrocession may be adduced. The pus secreted by the inflamed parts, and which heretofore concreted so as to form the inner surface of the crusts, may be transported to the membranes, or the small veins communicating between the interior and exterior of the cranium may propagate the inflammation to the pia mater; or these accidents may result from the state of congestion which hot topical applications have induced in the brain. To whichever of these causes the effect may be due, or if we admit retrocession, reason points out that the eruption must only be gradually healed, that too hot applications should not be used, and that a derivative action upon the intestinal canal should be maintained.

"If the explanation of these effects resulting from the disappearance of acute affections of the skin is difficult, how much more so is it in the case of one of a chronic nature. The time which intervenes between the disappearance of the one and the development of the other, and the possibility of the internal disease having arisen from so many different causes, generally render the solution of the problem impossible. Prudence teaches us that it is always better to act as if the possibility of the fact was admitted, and it is right to operate a revulsion upon another portion of the economy when endeavouring to relieve a cutaneous disease which has persisted for several years. So too, it may be necessary to favour the return of a cutaneous affection, when its disappearance is attended with a derangement of functions, which did not exist while it was present.

"It seems to us proper to avoid any active treatment in disease of the scalp, 1, when it succeeds to an obstinate ophthalmia, which has amended upon its appearance; 2, when, soon after the treatment has commenced, the eyelids and conjunctiva become injected, and an ophthalmia seems likely to occur; 3, when the development of the eruption has occurred in a very young and delicate child, whose general health seems sensibly improved since its appearance; 4, when the diminution of secretion is followed by general symptoms, however slight, such as loss of appetite, restlessness, moroseness, febrile action, &c."

*Erysipelas.*—The authors remark as a very singular fact that children, who are so liable to chronic inflammations of the face and scalp, are seldom the subjects of erysipelas of the face, and that in the cases which do occur it is very rare to observe cerebral complications. The erysipelas of new-born infants, generally commencing at the umbilicus, is a very fatal disease in hospitals.

*Cutaneous Diphtheritis.*—This curious affection, noticed by Bretonneau, has been especially described by Trousseau. It consists in a pseudo-membranous inflammation of the skin, prevailing during the epidemics of pseudo-membranous angina, affecting any part which becomes denuded of the cuticle, as by blisters, excoriation, friction, &c.

### *Induration of the Cellular Tissue.*

This is a common disease of new-born infants in France, but M. Bouchut states, he is quite unable to account for the great difference in the descriptions furnished by those who have written upon it. That by Billard he considers is nearest the truth. Sometimes the induration occupies the entire surface of the body, but at others only the limbs or the face. It is, in fact, an *œdema*, for more or less serosity is found in the cellular tissue, which M. Chevreul observed spontaneously coagulated when exposed to the air. The principal vessels of the parts affected are permeable and congested with blood, so as to produce great distension of the tissues. The cutaneous capillaries, however, are devoid of blood, and appear obliterated, although injection can be forced into them after death. The lungs are congested, and the skin frequently jaundiced, at other times reddish or of a dirty white, and very cold to the touch. The children suffer much and utter, from time to time, a shrill cry, like the hydrocephalic cry, but not so loud. The disease exists sometimes at birth, and at others comes on between the first and twelfth day. It continues from two to six days, and is fatal when very extensive. It cannot be confounded with the induration of the adipose substance, which takes place in some children just about to die, nor with the cadaveric induration of the same structure. The disease is seldom observed in other than in foundlings, and the children of the very poor. In its partial form it is now and then seen in older children and in adults.

### Class II.—Dropsies.

Accumulations of fluid in the cavities, and infiltrations into the cellular tissue, are very common affections of children, and, as in the adult, they may be either of an active or passive nature. Dropsies in children are however almost always but *secondary diseases*, and their acute or chronic nature, and degree of gravity, depend upon the nature of the primary affection producing them. As is the case with tubercle and inflammation, dropsical effusion may manifest itself simultaneously or consecutively in various organs.

The *Causes* of dropsies are thus enumerated :—

" 1. A sudden chill, or living in damp places, &c. The dropsy, whether general or local, is then often acute. 2. Inflammations of organs, producing active febrile dropsy, which is generally confined to the organ affected, although sometimes developing itself at another point. 3. Specific inflammations, and especially the eruptive fevers. Here the dropsy is hyperacute, acute, or cachectic, according to the period when it occurs. It is frequently general, or attacks a great number of organs at once. It is often dangerous. 4. Albuminuria. Under the influence of this cause the dropsy is sometimes hyperacute, oftener acute or chronic. Confined to the cellular tissue it may affect many organs, and is a grave disease. 5. Deterioration of the constitution by a disease of long duration, as tubercle, chronic disease of the digestive organs, intermittent fever, and in general all affections which produce an impoverishment and a defective plasticity of the blood. The dropsy is cachectic, occupying usually but one or part of an organ, and very rarely several organs. The disease producing it, not the dropsy, is the source of danger. 6. An obstacle to the venous circulation. This frequent cause of dropsy seldom exists alone in the child, but is accompanied by some serious disease predisposing to it, as hypertrophy of the heart, compression of the veins by tubercles, &c.

"In studying the causes we must not forget the influence of age and constitution; and we may repeat the remark we made respecting phlegmasiæ, that robust boys, aged above six years, are most liable to the acute active forms, while girls, and feeble children of less than six years old, are most liable to the chronic and cachectic forms.

"Several of these causes may unite in affecting the same individual. Thus a dropsy following an eruptive fever may likewise arise from albuminous nephritis; one caused by this last affection may also depend upon hypertrophy of the heart; while one arising from a tuberculous cachexia may likewise be due to venous compression."

### *Œdema of the Lungs.*

This is a very common affection in children, although, owing to the obscurity of the symptoms, not often detected, which is of the less consequence, as the primary disease is the real object of treatment. The effusion varies from a few drops to a very large quantity, pouring out on incision. In the 77 autopsies, in which it was observed by the authors, it was found generally to occupy the upper lobes. Secondary pneumonia and scarlatina were usually the producing diseases, and albuminuria frequently coexisted.

### *Hydrothorax.*

From the late period at which the symptoms often appear, or their entire absence, there is reason to believe the effusion (which may amount to a pint and more on each side), in many cases, occurs not long prior to death. Scarlatina and nephritis in the acute form, and tubercle in the cachectic form, were the usual primary diseases in the 36 cases examined by the authors.

The authors met with but six cases of augmented quantity of fluid in the *pericardium* (4 to 10 ounces) without accompanying marks of inflammation. The heart was however, in most, large and firm.

### *Ascites.*

When the quantity of fluid is small, it is often difficult of detection during life. The child should be held horizontally with its belly downwards, and the examination made near the umbilicus. In very young children, with large bellies, and especially if the parietes are œdematous, we may be often led to believe fluctuation exists when it does not. Retention of urine may be mistaken for ascites. In its acute form the disease has all the characters of secondary peritonitis, but after death the intestines are found pale and colourless. Albuminuria, scarlatina, and rubeola are the usual primary diseases. Of 25 autopsies, 20 occurred in boys, and 5 in girls, 7 in children less, and 18 more than six years of age. Paracentesis is rarely if ever advisable.

### *Hydrocephalus.*

This the authors define as a non-inflammatory effusion into the cranial cavities. They observe that it may be thought strange that they have devoted so little space to the consideration of the *acute form of the disease,* which usually occupies so much of the attention of writers on diseases of children. But they regard it as a mere epiphenomenon of *tubercular meningitis.* Without denying it may never be primary, they have never met with it but in a secondary form, as after nephritis, scarlatina, &c.

The changes which are operated upon the cranium and brain in the *chronic* form of the disease are set forth at length, but with these our readers must be well acquainted. The authors have never, in any form of the disease, been enabled to detect the sounds said by the American writers to be heard in auscultation of the cranium. Errors in diagnosis may arise—1, from a very small and thin face causing the head to appear proportionally large; 2, the thickening of the cranium by rickets. Upon examining the cranium this thickening will be found much more irregular than in hydrocephalus; 3, cerebral hypertrophy may cause an augmented size of the cranium. The authors speak very sceptically of the *curability* of this affection, and have no experience of the efficacy of puncture or compression. They have never seen it other than as a secondary affection, it having been generally consequent upon tuberculous tumours, which were so placed as to compress the sinuses or principal veins.

### *Anasarca.*

This is a common disease in children, and Rilliet and Barthez have met with it in one-eighth of the entire number of their patients. Only six cases, however, have been met with of the *primary* or idiopathic form. The *secondary* or *consecutive form* is sometimes general, in others partial. Of 155 cases it was acute in 79, and chronic in 76. The *acute* form usually comes on at an early stage of the primary malady, and is rapid and regular in its course. The *chronic* and *cachectic* form, occurring in those of feeble powers, or weakened by long illness, is variable and oscillatory in its progress. The primary disease may be an ordinary phlegmasia, inducing a plethoric condition of the general system, as pneumonia, pleurisy, angina, bronchitis. In such cases the dropsy is usually partial, acute, and of short duration. Local pressure upon the venous system may assist in these cases, and thus anasarca of the face, resulting from pressure of the vena cava superior by a pneumonia of the apex of the right lung, is not infrequent. One of the most frequent causes of acute or chronic anasarca, as already stated, is *nephritis,* which produces a severe and persistent form of the disease. But the most frequent cause of all, is *scarlatina,* and the other specific inflammations.

"Its mode of production is extremely complex, and explains to us why the infiltration is more frequent in certain specific affections than in others. Thus, in several of these diseases there exists a violent inflammation of the skin, which, as in erysipelas, causes considerable fluxion into the subcutaneous tissue. In that case the infiltration is truly active, very inflammatory, and accompanies the eruption. It is violent in proportion to the intensity of the eruption, and to it should be referred the active and painful subcutaneous swelling in variola, the general turgidity of scarlatina, and that, less considerable, of measles. But this immediate action of the skin on the cellular tissue is not the only one. The membrane seriously injured in its texture retains an anormal predisposition which acts for a long period in preventing the regularity of its functions. Hence result those dropsies consequent upon the eruptive fevers, and which manifest themselves especially at the period of desquamation. But on examining the nature of this predisposition, we discover that it is not the most violent eruption that is most often followed by anasarca, so that it cannot be the intensity of the cutaneous inflammation which determines the secondary infiltration. Variola, in fact, is hardly ever followed by anasarca, while scarlatina is so very frequently. We believe it is the seat rather than the intensity or nature of the inflammation which leads to this result. The seat of variola is the cutaneous follicles, that of rubeola the mucous body, or rather the superficial vascular network; while scarlatina appears to be an inflammation of the subcutaneous lymphatic tissue, whose functions are at once exhalant and absorbent. This is the cause of the anasarca; for, by reason of the lesion of the lymphatic tissue, the cutaneous exhalation is imperfectly performed, and upon its suppression by the access of even slight cold, an effusion results. If this occurs in rubeola or variola, it is because the cutaneous inflammation is sometimes propagated to the lymphatic tissue.

"This, then, is one of the causes of the anasarca in eruptive fevers. Add to this the deterioration produced by a general disease, and the evident change which occurs in the blood in these affections, as in all specific inflammations, of which a well-known result is a loss or diminution of its plasticity. Moreover, albuminous nephritis is frequently developed simultaneously with, or subsequently to, the original malady; while, in particular cases, various causes of obstruction of the circulation may also exist." 832.

Besides the causes of anasarca hitherto alluded to, we must notice a frequent one, viz. the *cachectic state,* induced by a severe or prolonged disease, attended with diminished plasticity of blood and apyretic passive effusion. It is usually partial, and frequently appears and disappears. Chronic gastro-enteric disease, ramollissement, tubercle, prolonged intermittents, &c., give rise to this form. The species and prevalence of anasarca seem, in some measure, occasionally to depend upon epidemic or seasonal influences.

As to the *age* most liable, of the 155 patients, 91 were less, and 64 more than six years of age. The *sexes* seem about equally affected, the boys being more liable to the acute, the girls to the chronic form.

The *treatment* of secondary anasarca will chiefly depend upon the nature and stage of the primary affection and the powers of the patients. Thus depletion, diuretics, especially digitalis, vapour-baths, aperients, or tonics, are indicated in different cases.

### Class III.—Hæmorrhages.

Hæmorrhages compared to dropsical effusions are of rare occurrence in the child, which may seem the more surprising, as they both arise from identical causes. The predominance of the lymphatic temperament explains the fact. Primary and active hæmorrhage hardly ever occurs, at least in very young children, passive hæmorrhage in the secondary form being that usually met with. The prognosis much depends upon the form. Primary hæmorrhage admits of cure in many cases. Acute secondary hæmorrhage is a much graver disease, especially if it occurs in several organs, and the primary affection is a specific one. It is of the worst possible augury when it occurs in the course of chronic or cachectic diseases. We may cursorily notice the varieties mentioned by Rilliet and Barthez.

1. *Hæmoptysis and Pulmonary Apoplexy.*—The former of these varieties of pulmonary hæmorrhage is very rare in children. The authors have met with but two cases of primary hæmoptysis, occurring in young girls, four cases occurring at a late stage of phthisis, and four accompanying gangrene of the lung. In 22 instances, *apoplexy of the lungs* has been observed. Most of the cases occurred in boys who had passed their fifth year, and the diseases which preceded it were tubercle, variola, scarlatina, nephritis, colitis, and secondary pneumonia.

2. *Epistaxis.*—Notwithstanding this affection in a slight degree is so frequent in children, the primary form in any excess is rarely met with, and even as a secondary disease it is much more rare than in the adult. It occurs chiefly in the course of eruptive fevers, typhoid and intermittent fever and hooping-cough. It is one of the gravest symptoms of purpura.

3. *Hæmatemesis* has never been observed by the authors in its primary form, and even in the cases recorded of its secondary form, they believe the organ has only acted as a receptacle for blood derived from other sources. Although much more frequent than this, *intestinal* hæmorrhage is yet a very rare affection, following eruptive and typhoid fevers, &c.

4. *Apoplexy.*—Of this the authors especially notice two forms, that in which the blood is effused into the arachnoid, and that in which it is effused into the substance of the brain. Among the causes enumerated are—1. Too violent treatment of diseases of the scalp. 2. Affections of the sinuses. 3. Obstruction of the vena cava superior by bronchial glands. 4. Compression of vessels by abdominal engorgements. 5. The cachectic state produced by prior maladies, diminishing the plasticity of the blood. 6. Occasionally, but very rarely, the disease is primary. Of *Meningeal Apoplexy* 17 cases were examined. The effused blood is represented as undergoing transformation into false membranes, sometimes resembling the arachnoid in texture, and at others being fibrous. When death occurs from cerebral tubercles in a child of two years old or less, bloody, not serous effusion, into the arachnoid is found. However common *cerebral apoplexy* is in the aged, it is an unimportant disease in the child, occurring as it does just before death, or in the course of mortal disease. Fourteen cases only are referred to, eight of which occurred to the authors. The paralytic symptoms, observed in the adult, are rarely seen in the child, and the disease is often latent, and its diagnosis seldom possible.

5. *Purpura.*—This affection, as in adults, exists in the two forms of *simplex* and *hæmorrhagica.* The former when primary, which it rarely is, is always curable, but when secondary, occurring as it generally does, in the course of severe chronic diseases, it is an indication of a lesion of the blood which presages their fatal termination. When the *hæmorrhagic* form is primary, recovery also generally occurs, but if constitutional, or secondary to other diseases, a very bad prognostic is to be entertained. The hæmorrhagic form is much more common in children who have passed their fifth year than in those who have not. Simple purpura is also found in much younger children. Epistaxis, buccal and intestinal hæmorrhages, are those which usually accompany it. Hæmaturia and hæmoptysis are much rarer. Eruptive fevers, and especially variola, are the most frequent primary diseases.

### CLASS IV.—GANGRENES.

Since these affections are almost always fatal in children, it is fortunate they are seldom met with. Any of the tissues of the economy may become affected, but the mouth, the pharynx, the lungs, and the skin, are the parts most liable. It is seldom that gangrenes exist alone, pneumonia, entero-colitis, or, though seldomer, tuberculization, usually accompanying them; nor is it rare to see several organs simultaneously gangrened. Gangrene is very rapid in its progress in the child, is never a primary disease, and chiefly affects the poor and miserable, who have been exposed to eruptive fevers, generally rubeola. The disease especially attacks children between three and five, and is sometimes endemic. Topical remedies, and an invigorating regimen, are usually indicated in its treatment.

1. *Gangrene of the Lungs.*—Sixteen cases of this are referred to, and the pathological anatomy described at length; but as this is the same as in the adult we need not notice it. Other lesions of the lung also commonly co-exist, as lobular pneumonia, carnification, œdema, &c. The diagnosis is usually difficult, the gangrenous odour of the breath being an important element in forming it.

2. *Gangrene of the Mouth.*—Twenty-eight cases form the foundation of the authors' observations, this being the most common site of the disease in children, to whom it is peculiar. It is a frightful affection, almost always mortal. In six cases the authors entered into a minute examination of the vessels of the gangrened parts. They found the walls of these to become somewhat thickened as they approached the swelling surrounding the gangrened parts, and plugged firmly with coagula as soon as they reached the latter. Of all coincident diseases pneumonia is the most common. It is remarkable that the children continue to eat heartily to the last. The distinction of this disease from *stomatitis* is chiefly formed by observing the absence of pseudo-membrane, the great and rapid extension of the ulceration, the accompanying hard engorgement, the abundant salivation, the eschar in the cheek or perforation of its substance, and destruction of the bones and teeth. The gangrenous odour too is more powerful. Rubeola is the most common antecedent disease, and it seldom follows scarlatina or variola. Free cauterization to some distance beyond the gangrened part offers the only chance of relief.

3. *Gangrene of Pharynx.*—This is a rare disease, for M. Bretonneau has proved, that the affection so frequently represented as gangrenous angina, was, in the great majority of cases, only a *pseudo-membranous angina,* the putrid false membranes being mistaken for eschars. This gangrene exists in a circumscribed or diffused form, and it usually appears at the termination of a secondary pneumonia, typhus, &c.; and other organs are often simultaneously affected.

4. *Gangrene of the Skin.*—This affection may shew itself in the form of what is termed *spontaneous gangrene* attacking the lower extremities and arising from a deposition of coagula in the arteries of the limb, which extend into the iliac and even the aorta; and *diffused gangrene,* which affects various portions of the surface, as the face, sacrum, vulva, &c. The latter may result from pressure, attacks the surface of blisters, or appears spontaneously—in all cases, however, coming on in the course of some other disease, especially the eruptive fevers. It was epidemic in the Children's Hospital at Paris, 1841.

We must defer the consideration of the remaining diseases until our next Number.

